# Lethal Interaction of Nuclear and Mitochondrial Genotypes in *Drosophila melanogaster*

**DOI:** 10.1534/g3.119.400315

**Published:** 2019-05-10

**Authors:** Tiina S. Salminen, Giuseppe Cannino, Marcos T. Oliveira, Päivi Lillsunde, Howard T. Jacobs, Laurie S. Kaguni

**Affiliations:** * Faculty of Medicine and Health Technology, FI-331014, University of Tampere, Finland; † Departamento de Tecnologia, Faculdade de Ciências Agrárias e Veterinárias, Universidade Estadual Paulista “Júlio de Mesquita Filho”, Jaboticabal, SP 14884-900, Brazil; ‡ Institute of Biotechnology, FI-00014, University of Helsinki, Helsinki, Finland; § Department of Biochemistry and Molecular Biology and Center for Mitochondrial Science and Medicine, Michigan State University, East Lansing, MI 48824

**Keywords:** cybrid, respiration, cytochrome *b*, mtDNA copy number, melanotic nodules

## Abstract

*Drosophila* *melanogaster*, like most animal species, displays considerable genetic variation in both nuclear and mitochondrial DNA (mtDNA). Here we tested whether any of four natural mtDNA variants was able to modify the effect of the phenotypically mild, nuclear *tko^25t^* mutation, affecting mitochondrial protein synthesis. When combined with *tko^25t^*, the mtDNA from wild strain KSA2 produced pupal lethality, accompanied by the presence of melanotic nodules in L3 larvae. KSA2 mtDNA, which carries a substitution at a conserved residue of cytochrome *b* that is predicted to be involved in subunit interactions within respiratory complex III, conferred drastically decreased respiratory capacity and complex III activity in the *tko^25t^* but not a wild-type nuclear background. The complex III inhibitor antimycin A was able to phenocopy effects of the *tko^25t^* mutation in the KSA2 mtDNA background. This is the first report of a lethal, nuclear-mitochondrial interaction within a metazoan species, representing a paradigm for understanding genetic interactions between nuclear and mitochondrial genotype relevant to human health and disease.

In virtually all eukaryotes, mitochondrial functions are maintained by a combination of genes located in two physically and functionally separate compartments: the nucleus and the mitochondrion. Mitochondrial DNA (mtDNA), typically present in many copies and inherited uniparentally, encodes only a small subset of the polypeptides required for the enzymatic functions of oxidative phosphorylation (OXPHOS). Because of maternal inheritance and the consequent lack of sexual recombination, combined with rapid segregation to homoplasmy in the germline, the mtDNA of a given individual may be considered as a haplotype.

In addition to 13 polypeptide subunits of OXPHOS complexes I, III, IV and V (cI, cIII, cIV, cV), involved in stepwise respiratory electron flow and ATP production, metazoan mtDNAs encode the RNA components (2 rRNAs and 22 tRNAs) of the separate translation system inside mitochondria. The remaining polypeptides of the OXPHOS complexes, as well as all others involved in mitochondrial metabolism, are nuclear-coded, and must be imported and assembled correctly inside the organelle. Because these functions are indispensible for life, mutations in either nuclear or mtDNA that impair OXPHOS or related processes, can result in drastic phenotypic consequences up to lethality. Examples may be found from the plant, fungal, protist and animal kingdoms. Nuclear and mtDNA may be considered to be a co-evolving system (Sloan 2015), with taxon-specific features themselves representing evolutionary adaptations to the environment (Otten and Smeets 2015).

In humans, OXPHOS mutations in both genomes have been widely studied because they are recognized as important genetic causes of disease. Because of the huge range of pathological phenotypes that characterize mitochondrial disorders, both within and between families, combined with the lack of a reliable model system, unraveling the precise contributions of nuclear and mitochondrial genotype to OXPHOS disease remains problematic. For example, one of the most common mtDNA-determined diseases, Leber’s Hereditary Optic Neuropathy (LHON) shows approximately fivefold greater penetrance in men than women. The attractive idea that this may be due to one or more X-chromosomal modifiers is broadly supported (Hudson *et al.* 2005; Shankar *et al.* 2008), although the precise loci involved have not been identified, and some studies have proven inconclusive with respect to specific populations, *e.g.*, Ji *et al.* (2010). Similarly, the role of mitochondrial haplotype as a phenotypic risk-factor or modifier of many pathological traits, such as neurodegeneration (Keogh and Chinnery 2015), has been extensively considered, but remains controversial. In most such instances, conclusions rest on statistical arguments regarding the size and nature of cohorts and the representativeness of population-based sampling.

In principle, because of strict maternal inheritance of mtDNA in most metazoans, the effects of different combinations of nuclear and mtDNA genomes can be studied experimentally by a back-crossing approach to create cybrid strains in which a given mtDNA haplotype is introgressed into different nuclear backgrounds. *Drosophila melanogaster* is a suitable organism for such studies because of its relatively short generation time (egg to sexually mature adult in ∼12 d at 25°) and the plethora of available, well-characterized strains and other genetic tools.

In two previous studies using this approach (Chen *et al.* 2012, Salminen *et al.* 2017), we detected subtle modifier effects upon organismal phenotype, of different mtDNAs present in wild populations of *D. melanogaster*. In a controlled, wild-type nuclear background (Oregon R, maintained long-term in the laboratory in Tampere, Finland, which we designate ORT), we found systematic effects of mtDNA on the time taken to complete development and on OXPHOS activities. These were found to correlate with mtDNA copy number (Salminen *et al.* 2017). In particular, long developmental time was associated with elevated copy number, suggesting a fitness cost of specific mtDNA haplotypes that could be compensated partially by increased mtDNA content.

In the other study (Chen *et al.* 2012), we introgressed different mtDNAs into a nuclear background bearing the *tko^25t^* mis-sense mutation (Shah *et al.* 1997) in the nuclear gene encoding mitoribosomal protein S12 (Royden *et al.* 1987). *tko^25t^* flies have significantly decreased levels and activities of all four OXPHOS complexes containing mitochondrial translation products (Toivonen *et al.* 2001), and exhibit a complex but mild organismal phenotype of developmental delay, mechanical stress-induced seizures, sensitivity to antibiotics and to high-sugar diet, and impaired hearing and courtship (Toivonen *et al.* 2001, Kemppainen *et al.* 2016). Four mtDNA backgrounds were found to confer a partial suppression of the *tko^25t^* phenotype (Chen *et al.* 2012).

In order to test for more dramatic manifestations of nuclear-mitochondrial interactions using this approach, we introgressed into the *tko^25t^* nuclear (n*tko^25t^*) background a set of mtDNAs from strains that were previously found to represent extremes of the spectrum of mtDNA copy number and developmental timing, when studied in the nuclear ORT (nORT) background (Salminen *et al.* 2017). The resulting cybrid strains revealed a clear-cut example of synthetic lethality between n*tko^25t^* and the mtDNA of strain KSA2 (mtKSA2), the first report of a lethal, nuclear-mitochondrial interaction within a metazoan species. This provides a paradigm for understanding genetic interactions between nuclear and mtDNA relevant to other contexts, including human health and disease.

## Materials and Methods

### Drosophila melanogaster strains and culture

*D. melanogaster* strains ORT, KSA2, WT5A, VAG1 (Salminen *et al.* 2017), and the *tko^25t^* mutant (Judd *et al.* 1972) were originally obtained from stock centers and maintained long-term on standard high-sugar diet (Kemppainen *et al.* 2016). Cybrid strains were derived as previously (Salminen *et al.* 2017) by repeatedly back-crossing females of a given strain to ORT males; then, in the case of *tko25t*, by back-crossing FM7-balanced nORT cybrid females of each mtDNA background to *tko25t* males; with a final cross to create homozygous *tko25t* cybrid females. Cybrid strains used in the experiments are designated nX mtY, where X is the specified nuclear and Y the specified mtDNA background. In controlled crosses to measure pupal stage phenotypes (mtDNA copy number, respiration and supercomplex activities), and adult stage phenotypes (eclosion timing and life span), *tko^25t^* cybrids were balanced over FM7, containing the Bar-eye marker. To distinguish *tko^25t^* from balancer larvae, the balancer also contained the dominant Tb marker (Bloomington line 36849, genotype FM7a, P{w[+mC]=Tb[1]}FM7-A).

### Egg-to-adult development and lifespan assay

Batches of 12 females and 6 males were mated and tipped 4 times at 24 h intervals, giving approximately 100-150 eggs per vial. Flies were reared at 25° on a 12 h light/dark cycle and 60% humidity, and eclosion was scored daily. To evaluate the effects of antimycin A treatment, flies were cultured on standard medium containing the sub-lethal dose of 5 μg/ml of the drug (Frei *et al.* 2005). Lifespan was measured using batches of 200 females and males transferred to fresh culture bottles every 2-3 d, with the number of dead flies recorded each time.

### Larval dissection

Wandering stage L3 larvae were dissected in a droplet of PBS under light microscopy, using thin forceps. Freely floating melanotic nodules were counted for each larva. Hemolymph was diluted into a total volume of 100 μl of PBS and hemocytes were counted using an Accuri C6 flow cytometer (BD Biosciences), with forward- and side-scatter gating used to distinguish total hemocyte count from debris (Anderl *et al.* 2016).

### Relative mtDNA copy number analysis

Total DNA was extracted from pools of 10 late-stage pupae of each sex, in four biological replicates, and copy number relative to a nuclear DNA locus was determined by quantitative PCR as described previously (Salminen *et al.* 2017). Briefly, mtDNA was quantified using primers against the 16S rRNA gene, and nuclear DNA using primers against the *RpL32* gene, based on calibration curves generated in parallel.

### Respiratory enzyme activities

Respirometry and blue-native electrophoresis (BNE) with in-gel histochemistry were performed as previously (Salminen *et al.* 2017), using homogenates from pupae collected soon after pupariation. A set of mtKSA2 nt*ko^25t^* pupal cases from this time point were dissected prior to the experiment, to verify that each contained a living individual, *i.e.*, to be sure that the assay was being conducted prior to the onset of lethality. Briefly, respirometry used a Clark-type electrode, with successive additions of standard substrates and inhibitors for the different respiratory chain complexes. BNE used the NativePAGE Novex Bis-Tris gel system (Invitrogen Life Technologies), with approximately equal loading checked by running aliquots of the samples on an SDS-PAGE gel that was then stained with Coomassie blue, prior to BNE. In-gel histochemistry was performed separately for cI and cIV, using standard staining procedures. Note that the blue dye used in BNE interferes with the in-gel assay for cIII, for which reason cIII was separately assayed enzymatically, using the same homogenates, as follows. Reduced decylubiquinone was prepared by adding a large excess of dithionite to a decylubiquinone solution (25 mg dissolved in 2.5 ml dimethyl sulfoxide plus 400 μl water, acidified by the addition of 50 μl 0.1 N HCl). The initially deep-orange solution was magnetically stirred for >30 min until it became transparent. After a full reduction (≥ 4 h) and the sedimentation of excess dithionite, the colorless upper phase was centrifuged (10,000 *g_max_*, 5 min) and the supernatant used in the assay. Ten μg aliquots of fly homogenates were added to a reaction mix containing 20 mM phosphate buffer, 100 μM cytochrome *c*, 20 μM KCN, 100 μM EDTA and 0.02% TWEEN 20 (pH 7.5), with and without the addition of antimycin A to 1 μM. After 3 min of incubation, 5 μl of reduced decylubiquinol was added and, after mixing, absorbance at 550 nm was followed at 21° for 2 min, at 10 s intervals. Complex III activity was inferred by subtracting the rate of absorbance change in the presence of Antimycin A from that in its absence.

### Molecular modeling

The structural effects of amino acid polymorphisms in *Drosophila* cytochrome *b* (cyt b) were evaluated by mapping onto the crystal structure of *Bos taurus* cIII, PDB 1BGY (Iwata *et al.* 1998), using the Pymol mutagenesis tool (www.pymol.org) under default settings. Pymol was also used to analyze all structural details and to produce the figures.

### DNA sequencing

Using the primers indicated in Table S1, the *Drosophil*a UQCR-C1 (CG3731) gene was amplified in two fragments by long PCR, using the same DNA preparations as for mtDNA copy number analysis, and then cycle-sequenced.

### Statistical analysis

mtDNA copy number variation and eclosion frequencies were analyzed by one-way ANOVA, combined with the *post hoc* Tukey HSD test (astatsa.com). Student’s *t*-test was used for pairwise comparisons of other data. Life-span data were analyzed using the Kaplan-Meier test (GraphPad Prism v7.00).

### Data Availability

Strains are available on request. The authors state that all data necessary for confirming the conclusions presented in the article are represented fully within the article. Supplemental material available at FigShare: https://doi.org/10.25387/g3.7796027.

## Results

### mtDNA variants modify the tko^25t^ phenotype

**Figure 1:**
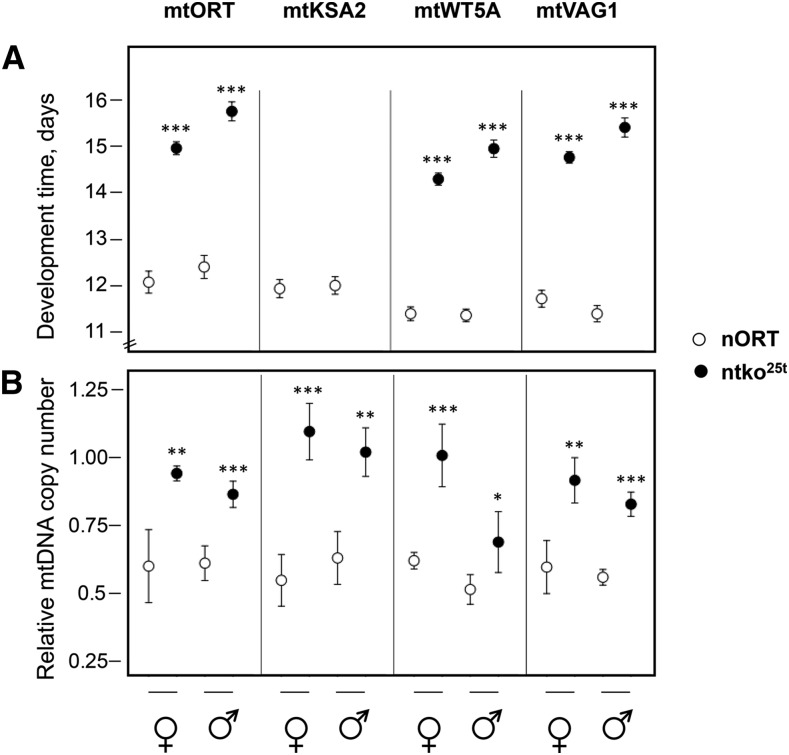
Developmental phenotypes of cybrid strains (A) Egg-to-adult development times of flies of the indicated sex and genotype. Note that the mtKSA2 haplotype was pupal lethal in the n*tko^25t^* background. (B) Late pupal mtDNA copy number, arbitrarily normalized to that of a wild-type ORT adult strain. All data plotted as means ± SD from at least four replicate vials. Asterisks (*, **, ***) denote statistically significant differences between nuclear backgrounds, for flies of a given sex and mtDNA haplotype (Student’s *t*-test, *P* < 0.05, 0.01, 0.001, respectively).

**Figure 2:**
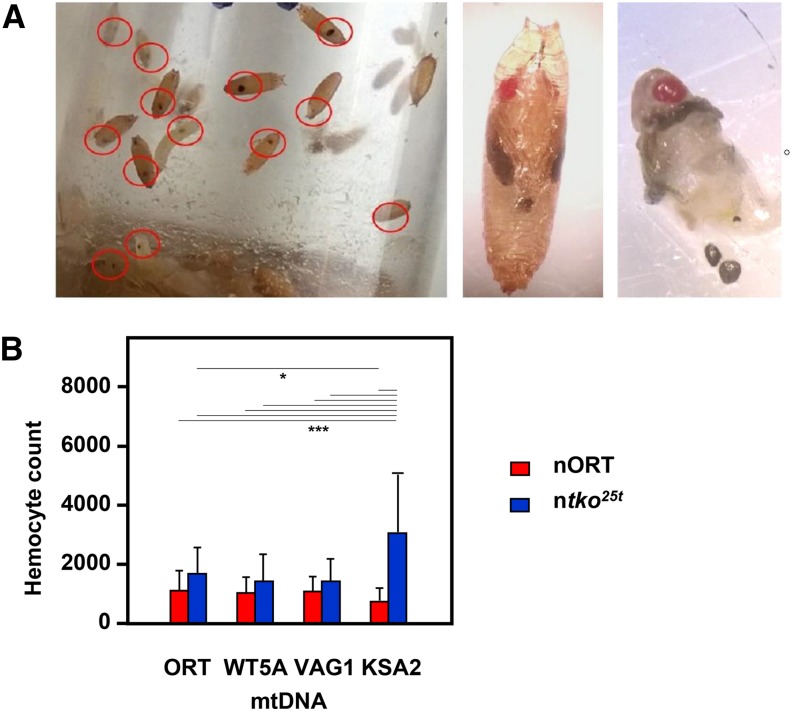
Hemolymph abnormalities in cybrid lines (A) Images of n*tko^25t^* mtKSA2 flies during wandering L3 and pupal stage. Red ellipses (left-hand panel) are drawn around an example of melanotic nodules. After dissection (right-hand panel), most nodules were observed to be free of association with any specific tissue. (B) Total hemocyte counts from hemolymph of individual L3 larvae from indicated cybrids (means ± SD of at least 15 larvae of each genotype). Results of ANOVA followed by Tukey *post hoc* HSD test indicated by horizontal lines joining values showing significant differences (95% CI, *, *** denoting *P* < 0.05 or < 0.001 respectively).

To investigate genetic interactions between mtDNA and the nuclear *tko^25t^* mutation, which affects mitochondrial protein synthesis, we introgressed four variant mtDNAs into both the wild-type (nORT) and n*tko^25t^* nuclear backgrounds. We then assessed the effect of different mitonuclear combinations on development at 25°. In the nORT background, the egg-to-adult development time was ∼12 days in all cases (Figure 1A), whereas n*tko^25t^* flies exhibited a developmental delay of 3-4 days, which was slightly more pronounced in males (Figure 1A). In the n*tko^25t^* mtKSA2 cybrid, larvae pupariated approximately three days later than in other n*tko^25t^* cybrid strains and failed to eclose. For the other 3 mtDNAs tested, the developmental delay in the *tko^25t^* nuclear background was very similar (Figure 1A). Nuclear and mtDNA backgrounds had only minor effects on longevity (Figure S1). Most n*tko^25t^* mtKSA2 pupae contained melanotic nodules (Figure 2A), which were also evident in dissected L3 larvae. Such nodules are considered a visual marker of dysregulated innate immunity. The frequency of such nodules was co-determined by the nuclear (n*tko^25t^*) and mitochondrial (mtKSA2) genotypes (Table 1), and their presence was accompanied by an increased number of hemocytes (Figure 2B) in n*tko^25t^* mtKSA2 larvae compared with the nORT (control) nuclear background. Based on the fact that mtKSA2 produced lethality and melanotic nodules in combination with n*tko^25t^*, we focused the remainder of the study on this strain, for which the other three mtDNA haplotypes can be considered, essentially, as controls.

**Table 1:** Frequency of melanotic nodules in L3 larvae of cybrid strains

mtDNA genotype	% larvae^1^ with nodules in nORT background	% larvae^1^ with nodules in n*tko^25t^* background	% larvae^2^ with nodules in nORT background + 5 μg/ml atimycin A
mtORT	0	0	24
mtKSA2^3^	6	56	(lethal)
mtVAG1	0	1	33
mtWT5A	0	1	25

1 n = 150 in each case.

2 n = 100 (mtORT), 61 (mtVAG1) or 45 (mtWT5A)

3 13% of the larvae (n = 150) of the original parental strain KSA2 (*i.e.*, mtKSA2, nKSA2 using the same nomenclature) also showed at least one melanotic nodule, in the absence of antimycin A treatment.

### mtDNA copy number is elevated in ntko^25t^ in all mtDNA backgrounds tested

Because our earlier studies implicated mtDNA copy number as a relevant marker of phenotype conferred by mtDNA background, we investigated whether this might correlate with and explain the lethality of the mtKSA2 n*tko^25t^* combination. Because mtKSA2 was shown previously to confer lower mtDNA copy number in adults (Salminen *et al.* 2017), we studied the latest developmental stage at which n*tko^25t^* mtKSA2 flies could be evaluated (late pupae). Copy number (Figure 1B) was broadly similar between mtDNA haplotypes in the nORT background, whereas n*tko^25t^* cybrids showed a significantly elevated copy number compared with nORT in both sexes in all mtDNA backgrounds, including mtKSA2. This appeared to be highest of all in the case of the lethal mtKSA2 n*tko^25t^* combination, although the observation may be affected by the fact that these pupae were destined to die prior to eclosion, and therefore may not be strictly equivalent, developmentally, to those of the other strains. Copy number elevation appeared to be modest in mtWT5A n*tko^25t^* males (Figure 1B), although this was not correlated with any observable developmental difference from the other strains.

### Antimycin A phenocopies effects of ntko^25t^ on mtKSA2 flies

**Figure 3:**
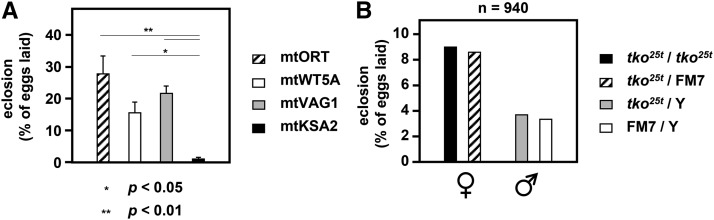
Eclosion frequency of nORT cybrids cultured on medium containing antimycin A (A) Egg-to-adult eclosion frequency (*i.e.*, percentage of eggs completing development and emerging as adults) of the indicated cybrid lines, cultured in 5 μg/ml antimycin A. Note that all emerging flies in a given culture were of the same genotype. Means ± SEM of 7 or 8 replicate vials (∼65 eggs laid per vial). Significant differences based on one-way ANOVA with *post-hoc* Tukey HSD test. The dose of antimycin was based on preliminary trials to confirm the sub-lethal concentration inferred from the literature (Frei *et al.* 2005). (B) Egg-to-adult eclosion frequency for progeny flies of each genotype indicated, in the cross *tko^25t^* / FM7 x *tko^25t^* / Y, cultured on medium containing 5 μg/ml antimycin A. The flies used in the cross were all in the control mtDNA background mtWT5A. Note that males were more severely affected by the drug than females (which was observed for all genotypes tested), but the proportion of flies of a given sex eclosing on antimycin A was independent of *tko* genotype (chi-squared test, *P* > 0.05 for both sexes). Data are from a single, large-scale experiment (n = 940) of sufficient size to generate statistically robust values.

The n*tko^25t^* mutation has a global effect on mitochondrial protein synthesis, decreasing the activity of all three of the respiratory chain complexes, dependent on mitochondrial translation products (Toivonen *et al.* 2001, 2003). We therefore set out to test whether the synthetic lethality of n*tko^25t^* with mtKSA2 could be explained by its effect on respiration, by culturing mtKSA2 flies in the control (nORT) nuclear background on medium containing a sub-lethal dose of a respiratory chain inhibitor, antimycin A. Based on trial experiments with wild-type lines, we confirmed 5 μg/ml as a suitable concentration of antimycin A in fly food, yielding semilethality (an egg-to-adult eclosion of approximately 30%). Exposure to antimycin A was lethal in combination with mtKSA2, but not the other three mtDNA haplotypes tested (Figure 3A). The lethality occurred mostly at an early stage, producing too few wandering stage L3 larvae to assess the presence of melanotic nodules. In mtDNA backgrounds other than mtKSA2, ∼25% of nORT L3 larvae had melanotic nodules, when cultured on antimycin A-containing medium (Table 1). We also observed that mtKSA2 was larval-lethal in the presence of antimycin A in its original nuclear background. In the converse experiment, antimycin A treatment was not lethal to n*tko^25t^* flies in a control mtDNA background (Figure 3B), with no n*tko^25t^*-specific effects on eclosion frequency compared with balancer controls.

### mtKSA2 confers a specific defect in respiratory complex III

**Figure 4:**
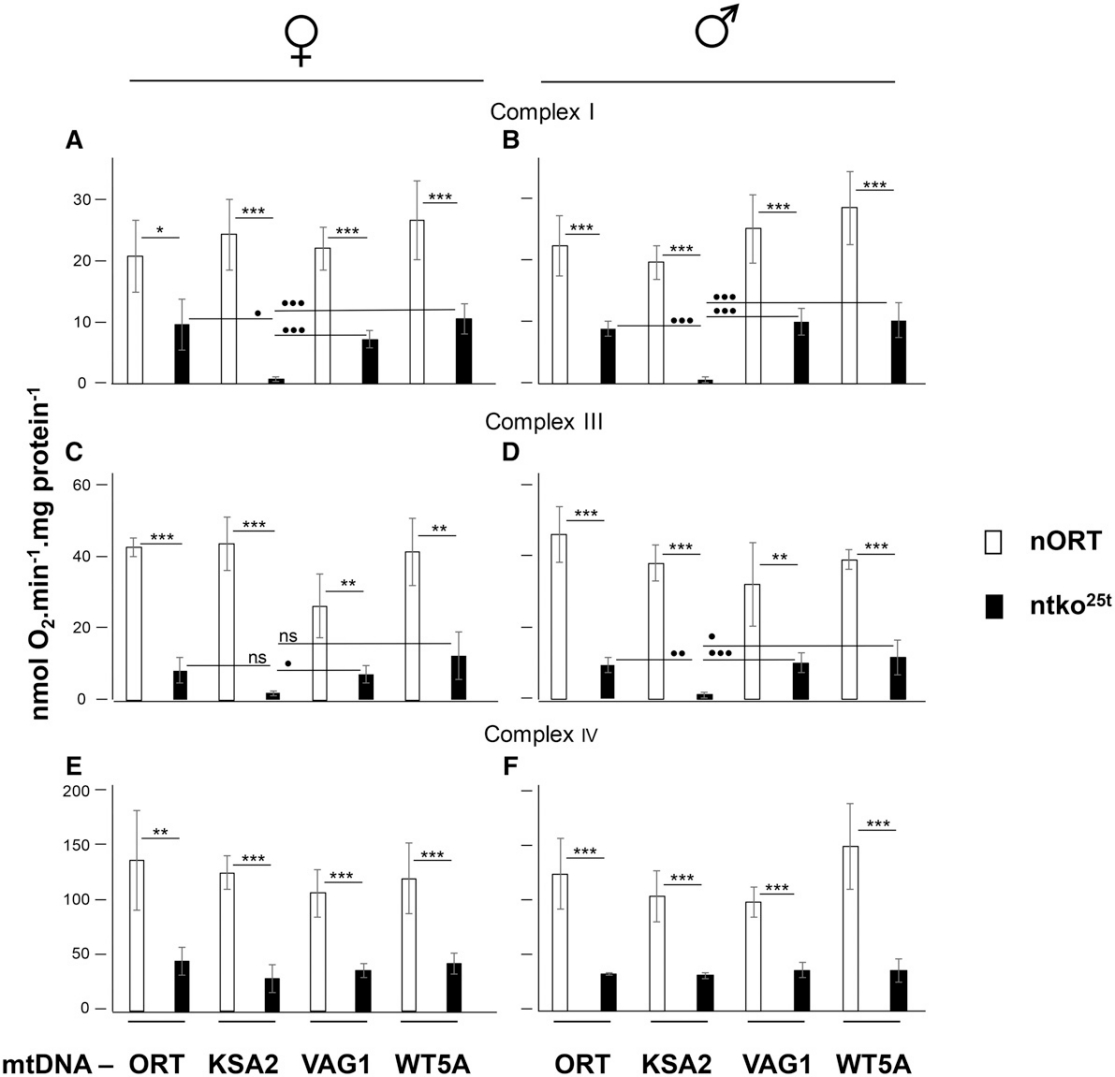
Respirometry of homogenates from cybrid strains State 3 respiration on cI-, cIII- and cIV-linked substrate mixes, of homogenates from pupae of the sex and genotypes indicated. Asterisks above the bars (*, **, ***) indicate significant differences between the nuclear backgrounds (Student’s *t*-test, *P* < 0.05, 0.01, 0.001, respectively); filled circles (•, ••, •••) denote significant differences between mtDNA haplotypes for a given nuclear background (Bonferroni-corrected Student’s *t*-test, *P* < 0.05, 0.01, 0.001, respectively; ns – not significant).

**Figure 5:**
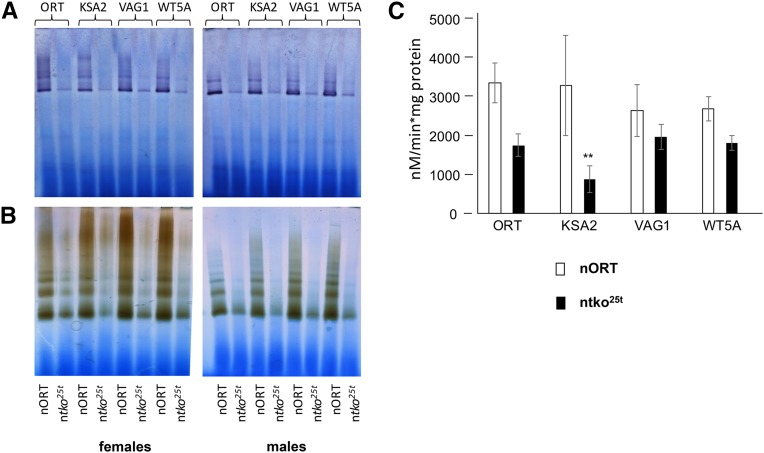
Respiratory activities in pupal homogenates of cybrid strains (A, B) Blue-native electrophoresis and in-gel histochemistry for (A) cI, (B) cIV, of strains of the indicated mtDNA haplotypes in the nORT and n*tko^25t^* backgrounds as shown. (C) cIII assay (means ± SD of four biological replicates) from strains containing the indicated mtDNAs in the nORT and n*tko^25t^* backgrounds as shown. ** denotes statistically significant differences between the nuclear backgrounds for a given mtDNA haplotype (Student’s *t*-test, *P* < 0.01).

Having established that the effects of n*tko^25t^* in combination with mtKSA2 can be phenocopied by the presence of a respiratory chain inhibitor, we set out to test how mtKSA2 influences respiratory activity in both the n*tko^25t^* and control (nORT) backgrounds. The coding-region of mtKSA2 mtDNA has two unique amino acid replacements compared with the reference sequence (Salminen *et al.* 2017), one in the Cox3 subunit of cIV (A75T), the other in cytochrome b (D21N), which is the only mtDNA-encoded subunit of cIII. In contrast, the mtDNAs of the control strains ORT, WT5A and VAG1 contain no nonsilent changes in protein-coding sequences other than those shared with other members of their haplogroup (Salminen *et al.* 2017). To test whether the unique nonsilent changes in mtKSA2 mtDNA produce a respiratory defect that could explain synthetic lethality with n*tko^25t^*, we measured mitochondrial oxygen consumption in homogenates of pupae from nORT and n*tko^25t^* cybrid strains. Respiration of all the n*tko^25t^* cybrids was significantly lower than the corresponding nORT cybrids on cI-, cIII or cIV-linked substrate mixes (Figure 4A-4F), in accordance with previous findings (Toivonen *et al.* 2001, 2003). For n*tko^25t^* mtKSA2 pupae, respiration using both cI- and cIII-linked substrate mixes was lower still (Figure 4A-4D: note that these measurements are surrogates for complex I+III+IV and complex III+IV activity, respectively, and not for the individual complexes). Respiration on the cIV-linked substrate mix was uniform among the four *tko^25t^* cybrid strains, in either sex (Figure 4E, 4F). Blue native electrophoresis combined with in-gel histochemistry showed decreased activity of cI and cIV in all n*tko^25t^* cybrids, compared with nORT cybrids, with greatly decreased amounts of detectable supercomplexes, but no specific effect attributable to mtDNA haplotype (Figure 5A, 5B). In contrast, the activity of cIII was significantly lower in n*tko^25t^* mtKSA2 cybrids than in the other mtDNA haplotypes tested (Figure 5C).

### Molecular modeling indicates specific destabilization of subunit interactions in complex III by mtKSA2 variants

**Figure 6:**
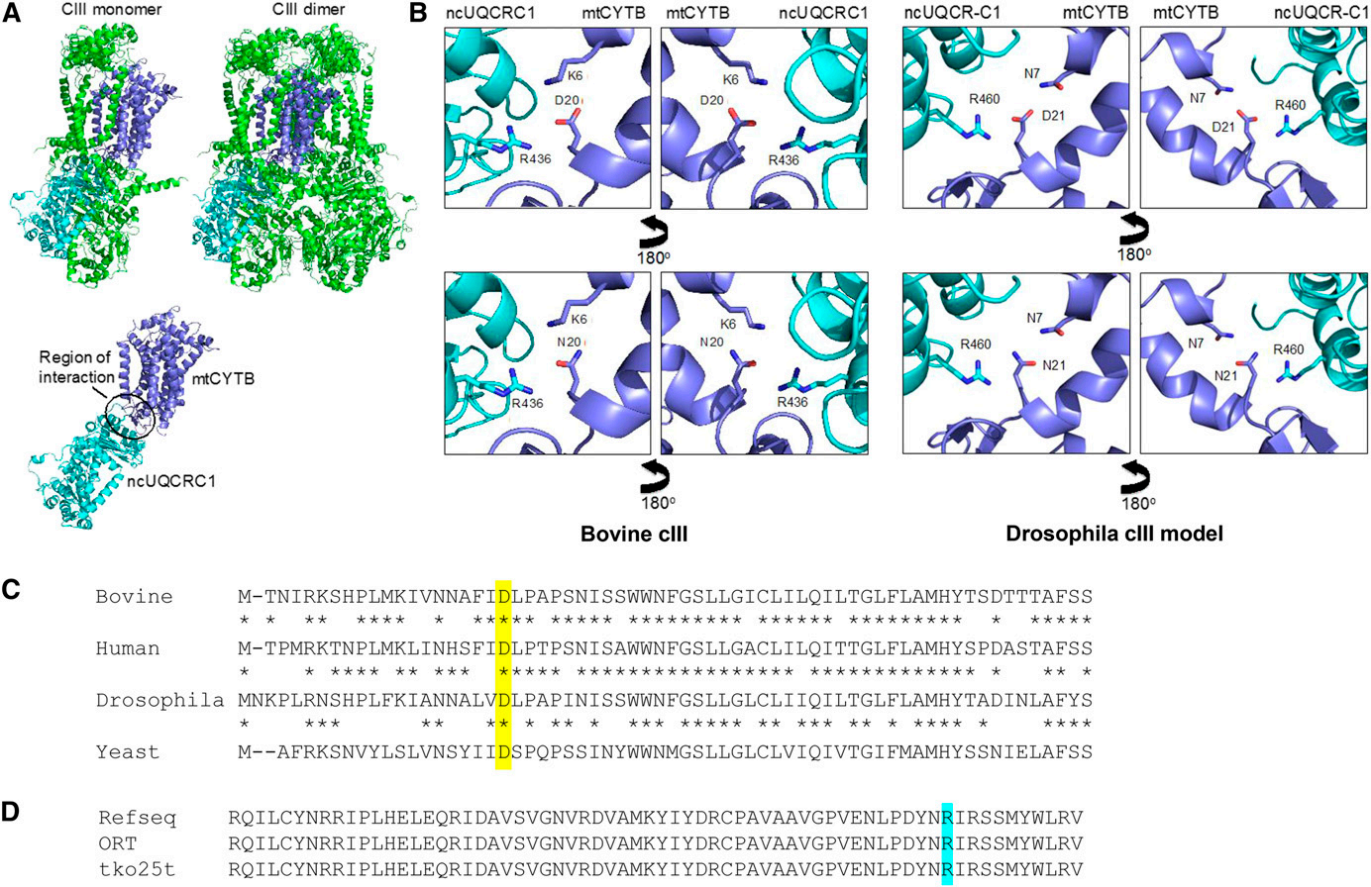
Molecular modeling and sequence alignments (A) Crystal structure of bovine cIII, showing the monomer, dimer and subunit interface between cytochrome *b* (mtCYTB, dark blue) and core 1 (UQCRC1, light blue). Other subunits shown in green. (B) Details of the structure, showing conserved residue D20 of mtCYTB, and its proximity contacts with other amino acids. In the bovine structure, D20 of mtCTYB interacts electrostatically with R436 of UQCRC1 and with K6 of mtCYTB. In *Drosophila*, K6 is replaced by N7, which is still able to interact with D21 (equivalent to bovine D20) via a hydrogen bond, whereas the charged groups of D21 and R460 (replacing bovine R436) should interact electrostatically. The D21N replacement would abolish this electrostatic interaction (the amino groups of N21 and R460 may even repel), weakening the overall structure, and potentially interfering with the complex reaction mechanism of the enzyme. (C) Partial amino acid alignment of the cytochrome *b* polypeptides of the four species, indicated (Bovine – *Bos taurus*, NCBI AAZ95348, commencing with M1; Human – *Homo sapiens*, NCBI ADT79912; Drosophila – *D. melanogaster*, NCBI AAA69714; Yeast – *Saccharomyces cerevisiae*, NCBI CAA24073. The conserved aspartate that is replaced by an asparagine in mtKSA2 is shown against yellow background. (D) Partial sequence of UQCR-C1 polypeptide of *D. melanogaster* (Refseq – NCBI Q9VFF0, commencing at R405; inferred sequences for strains ORT and *tko^25t^*, based on DNA sequencing). The conserved arginine involved in interactions with cytochrome *b* is shown against turquoise background.

Having established that mtKSA2 has a drastic effect on complex III activity in the n*tko^25t^* background, in which mitochondrial translation products are limiting, we next asked if this could be due to the structural effects of the unique coding-region mutations present in KSA2 mtDNA (Salminen *et al.* 2017). To address this question, we carried out molecular modeling (Figure 6A, 6B). Based on the crystal structure of *Bos taurus* cIII (Iwata *et al.* 1998), the *Drosophila cyt b* D21N mutation (D20N in the bovine sequence) is predicted to disrupt an ionic interaction with R460 of the nuclear-coded core 1 subunit (UQCR-C1) (Figure 6B). Sequence alignment of the cyt *b* polypeptide between human, cow, *Drosophila* and yeast (Figure 6C) shows that D21 lies at the beginning of a conserved sequence block. We confirmed the presence of R460 in the predicted UQCR-C1 polypeptide of both ORT and n*tko^25t^* strains, by partial sequencing of the gene (Figure 6D). The cIV Cox3 subunit in which the A75T mutation lies is one of several that make contacts with cIII, based on the structure of the yeast (cIII)2(cIV)2 supercomplex (Heinemeyer *et al.* 2007). However, there are too many uncertainties regarding the overall structure of these supercomplexes in *Drosophila* to model the precise effect of A75T, which in any case is less highly conserved phylogenetically than D21 of cyt *b*.

## Discussion

In this study we found that two relatively mild mutations in *Drosophila melanogaster*, respectively in nuclear and mitochondrial DNA, combined to produce developmental lethality. This is the first report of a lethal, nuclear-mitochondrial interaction within a metazoan species, and represents a paradigm for understanding interactions between the two genomes more generally, notably in human health and disease. Based on the functional interaction of mtKSA2 with antimycin A (Figure 3), together with respirometry (Figure 4), BNE in-gel histochemistry (Figure 5A, 5B), enzymology (Figure 5C) and molecular modeling (Figure 6), we are able to propose a plausible molecular mechanism underlying the synthetic lethality of mtKSA2 and n*tko^25t^*. A modest enzymatic defect in cIII becomes functionally critical when combined with a mitochondrial protein synthesis deficiency that affects globally the respiratory chain.

### Precedents for nuclear-mitochondrial incompatibility

Nonlethal mitonuclear interactions within a species have been previously reported in *Drosophila* by ourselves (Chen *et al.* 2012) and others, such as the influence of mtDNA haplotype on a nuclear mutant affecting cI (Loewen and Ganetzky 2018). Adaptive co-evolution of nuclear and mitochondrial DNA has also been demonstrated in nematodes, both in the wild (Chang *et al.* 2015) and in the laboratory (Wernick *et al.* 2019). In contrast, while overt nuclear-mitochondrial incompatibility is well documented in yeast (Zeyl *et al.* 2005) and plants (Ogihara *et al.* 1999, Gabay-Laughnan *et al.* 2009, Barr and Fishman 2010), it has been previously reported in metazoans only in cases of inter-species hybrids (Nagao *et al.* 1998, Kwon *et al.* 2016, Ma *et al.* 2016), where it has been proposed to contribute to speciation (Hill 2016). For example, maternal effect lethality results from incompatibility between the nuclear-coded, mitochondrial tyrosyl-tRNA synthetase of *D. melanogaster* and the mtDNA-encoded tRNA^Tyr^ of *D. simulans* (Zhang *et al.* 2017). The present finding differs from these examples because lethality is intraspecific, but involves both a laboratory-isolated mutant (*tko^25t^*) and a natural population (KSA2). While its applicability to population biology may thus be limited, it clearly has potential relevance to human disease, as discussed below.

### Molecular basis of synthetic lethality

The decreased respiratory activity seen in all n*tko^25t^* cybrid lines is consistent with previous data (Toivonen *et al.* 2001, 2003). It reflects the fact that the n*tko^25t^* mutation results in decreased abundance of mitoribosomal small subunits (Toivonen *et al.* 2001), leading to decreased capacity for mitochondrial protein synthesis. In turn this affects all four OXPHOS complexes dependent on mitochondrial translation products, *i.e,*. complexes I, III, IV and V. In the present work, we found that mtKSA2 confers an additional decrease of complex I+III+IV and III+IV activities (Figure 4), as well as isolated enzymatic activity of complex III (Figure 5B). In contrast, the activities of cI and cIV, based on BNE gel plus in-gel histochemistry (Figure 5A, 5B) were affected to a similar extent in mtKSA2 and control n*tko^25t^* cybrids. These findings identify cIII as the OXPHOS complex most impaired by the KSA2 mtDNA haplotype, and are consistent with molecular modeling, which indicated the likelihood of impaired subunit interactions within cIII (Figure 6), and possibly between cIII and cIV. An additional effect on electron channeling between cIII and cIV might explain why the combined respiratory activities (Figure 4) were proportionately more affected by mtKSA2 than isolated cIII (Figure 5C). Treatment with a moderate concentration of the respiratory inhibitor antimycin A was synthetically lethal with mtKSA2, although not with n*tko^25t^*. Thus, the drug appears to phenocopy effects of n*tko^25t^*, and the synthetic lethality of mtKSA2 with n*tko^25t^* can be explained by the combined effect of decreased cIII activity conferred by the former, and the global impairment of respiration resulting from the mitochondrial protein synthesis defect of the latter. Because antimycin A, which also inhibits cIII, did not show interaction with n*tko^25t^* in a control mtDNA background, we infer that the impairment of cIII activity resulting from the *tko^25t^* mutation is not rate-limiting for respiration in such a background. This inference is consistent with studies showing that by-pass of cIII+IV using the alternative oxidase had no effect on the *tko^25t^* phenotype, whereas the alternative NADH dehydrogenase Ndi1 was deleterious (Kemppainen *et al.* 2014), implicating cI as the complex most affected in *tko^25t^* .

### Modulation of mtDNA copy number

On the basis of previous data (Salminen *et al.* 2017), we proposed that programmed adjustments to mtDNA copy number could compensate for decreased fitness, and that respiratory deficiency under stress conditions may activate a sensor mechanism to generate such a response. This is consistent with the elevated mtDNA copy number seen in the n*tko^25^*^t^ cybrid strains studied here. Moreover, it was highest in n*tko^25t^* mtKSA2 pupae (Figure 1B), which also showed a clear respiratory defect (Figures 4, 5). Because the defect is associated with functionally significant coding-region mutations in KSA2 mtDNA, elevated copy number is most likely a consequence of respiratory stress rather than its cause, but any compensatory effect of copy number increase was insufficient to overcome lethality. By contrast, adult mtKSA2 flies were observed previously to manifest a *low* mtDNA copy number in wild-type nuclear backgrounds, despite manifesting relatively slow development (Salminen *et al.* 2017).

### Significance of melanotic nodules in mtKSA2 larvae

Oxidative stress in the posterior signaling center of the lymph gland is known to potentiate cytokine signaling that leads to overproduction of lamellocytes (Sinenko *et al.* 2012), the blood cells responsible for encapsulating and killing parasitic wasps (Rizki and Rizki 1992). Even in the absence of wasp infection, this can manifest as melanotic nodules (Sinenko *et al.* 2012), as also seen in mtKSA2 n*tko^25t^*, or in wild-type flies cultured in antimycin A medium (Table 1). The observed increase in total hemocyte count (Figure 2B) is part of the same response (Hanratty and Dearolf 1993, Qiu *et al.* 1998). Although the response can be elicited by other means, such as aberrant Toll (Hanratty and Dearolf 1993) or JAK/STAT signaling (Qiu *et al.* 1998, Harrison *et al.* 1995), by chromatin alterations (Braun *et al.* 1998, Walker *et al.* 2011), or even just by cuticular wounding (Márkus *et al.* 2005), the observed blood cell phenotype in mtKSA2 flies is also consistent with a primary respiratory chain defect, such as achieved elsewhere by knockdown of cI or cIV subunits (Sinenko *et al.* 2012).

### Spread of a deleterious mtDNA mutation

mtKSA2 flies harbor a potentially deleterious mtDNA genotype, conferring catastrophic consequences in specific nuclear backgrounds or when exposed environmentally to OXPHOS poisons. Thus, its presence in the KSA2 strain requires explanation. First, in the nuclear background in which it arose (Salminen *et al.* 2017), as well as in other ’wild-type’ nuclear backgrounds such as nORT (Salminen *et al.* 2017 and Figure 1), mtKSA2 is apparently neutral with respect to developmental timing and other traits, including cIII activity. Thus, its presence could simply be accounted for as genetic drift. Second, although lethal in the presence of high doses of antimycin A (which would be encountered only rarely in nature), the potentiation of lamellocyte differentiation in response to milder stress, as represented in hemocyte proliferation and the propensity to form melanotic tumors, is consistent with a status primed to resist parasitic infection (Eslin and Prévost1998). Fly strains are already known to vary with respect to susceptibility to such infection (Kraaijeveld and Godfray 1999, Gerritsma *et al.* 2013), and we speculate that mtDNA genotype could be a factor that predisposes to resistance. Parasite resistance seems particularly important in certain environments (Kraaijeveld and Godfray 1999), and may outweigh any negative effects of decreased OXPHOS efficiency, especially if nutritional resources are plentiful.

Positive selection is less of a consideration for n*tko^25t^*, which arose as a result of a mutagenic screen (Judd *et al.* 1972), and has not been seen in wild populations. Moreover, it is associated with a courtship defect (Toivonen *et al.* 2001). However, the net effect of the latter on survival in the wild is not clear, because the mutation appears simultaneously to decrease male attractiveness and female discrimination (Toivonen *et al.* 2001). Nevertheless, *tko^25t^* is also a phenotypically mild mutation. Because mtDNA mutations affecting the mitochondrial translation system appear largely to escape purifying selection (Stewart *et al.* 2008) it is possible that genetic variants with similar effects to *tko^25t^* could exist in natural populations. It has been argued elsewhere (Hill 2018) that mitonuclear selection could play a role in mate choice, and *Drosophila* may be a convenient organism in which to test this hypothesis.

### Relevance to human populations and disease

Our previous observation of mtDNA haplotypes acting as partial suppressors of n*tko^25t^* (Chen *et al.* 2012), combined with the present findings of synthetic lethality with a different mtDNA background, offer a potential paradigm for understanding mitochondrial genetics more widely, including in humans. ’Mitochondrial genotype’, regarding both nuclear and mtDNA, is not simply a case of good *vs.* bad. As here, it can specifically affect one or more OXPHOS complexes, can influence ROS production, signaling, mitochondrial heat production, substrate utilization and even tissue-specific phenotypes linked to any of these. Mitochondrial disease is known to exhibit sharp threshold effects (Rossignol *et al.* 2003), and for overt pathology to be precipitated by commonly experienced external stresses, such as viral infection (Schapira 2000, Parikh *et al.* 2013), which also impact mitochondrial function (Scholte 1988). Thus, nDNA/mtDNA combinations that are entirely viable in one environment may be pathological or even lethal in another. Although *tko^25t^* was a laboratory-generated mutant, disease-causing mutations in mitoribosomal protein genes have been reported in humans (Saada *et al.* 2007, Lake *et al.* 2017, Borna *et al.* 2019), almost always as homozygotes or compound heterozygotes. It is likely that milder mutations affecting the mitoribosome are segregating naturally in the human population which, in combination with an equally mild mutant mtDNA equivalent to mtKSA2, which was isolated by chance from the wild, could cause a severe pathological phenotype.

Our findings thus provide a concrete example of highly deleterious mitonuclear interactions, and argue that the issue needs to be taken into account in considering the possible effects of mtDNA as a pathological co-factor or modifier (Chou and Leu 2015), as well as the advisability of donor-recipient matching in mitochondrial replacement therapy (Reinhardt *et al.* 2013, Wolf *et al.* 2015). Naturally arising mitonuclear incompatibilities have recently been proposed as a driver of selection during human evolution (Sharbrough *et al.* 2017). However, the complex genetics inherent to the n*tko^25t^* mtKSA2 combination implies that similar examples in the human population may be hard to recognize, and may have been overlooked, especially given the profusion of rare mtDNA variants (Kim and Schuster 2013).
